# Long-term follow up of malignant transformation of epidermoid cyst at the cerebellopontine angle based on serial imaging findings; A case report and literature review

**DOI:** 10.1007/s13691-026-00853-7

**Published:** 2026-03-03

**Authors:** Koji Saito, Akihiko Teshigawara, Miku Maeda, Nei Fukasawa, Yasuharu Akasaki, Yuzuru Hasegawa, Masayuki Shimoda, Yuichi Murayama, Toshihide Tanaka

**Affiliations:** 1 Department of Neurosurgery, The Jikei University School of Medicine, Kashiwa Hospital, Kashiwa-si, Japan; 2 Department of Neurosurgery, The Jikei University School of Medicine, 3-25-8 Nishi-Shimbashi, Minato-ku, Tokyo, 105-8461 Japan; 3 Department of Pathology, The Jikei University School of Medicine, Tokyo, Japan; 4 Department of Pathology, Moriyama Memorial Hospital, Tokyo, Japan

**Keywords:** epidermoid cyst, malignant transformation, squamous cell carcinoma

## Abstract

We report a case of malignant transformation of epidermoid cysts during long-term follow up more than 10 years with literature review. Epidermoid cysts are benign congenital tumors, accounting for 0.2–1.8% of intracranial tumors. Malignant transformation is extremely rare, with limited reports describing the imaging features and prognosis. A 69-year-old woman had presented with a tumor exhibiting hyperintensity on diffusion-weighted imaging at the left cerebellopontine angle and had been followed-up for the past decade. After 10 years, she developed left facial paralysis and hearing impairment. Contrast-enhanced lesions appeared within the tumor, accompanied by edema. Intraoperative findings revealed a tumor with two distinct components of pearly tumor and hematoma invading the cranial nerves. The pathological diagnosis was squamous cell carcinoma without primary malignancies, suggesting malignant transformation of epidermoid cyst. Although the residual tumor initially seemed dormant after postoperative radiotherapy, the patient developed carcinomatous meningitis. The literature was searched for squamous cell carcinoma without primary cancers showing neuroradiological changes in signal intensity on diffusion- and T2-weighted imaging, revealing 103 cases with a mean interval of 106.1 months from initial diagnosis to malignant transformation of epidermoid cyst. Although postoperative radiotherapy or chemotherapy has been attempted, no standard has been established for adjuvant treatment. The clinical benefits of postoperative adjuvant therapy need to be confirmed in further studies. As neuroradiological alterations on diffusion-weighted imaging or Gd-enhanced T1-weighted imaging during follow-up of epidermoid cyst might indicate malignant transformation of epidermoid cyst, postoperative radiotherapy might be an option worth exploring.

## Introduction

Epidermoid cyst (EpiC) is generally considered a benign tumor arising from remnant fetal tissue (0.2–1.8% of intracranial tumor) [1]. And cholesteatoma is also known as epidermoid cyst especially in involving the petrous bone and the intracranial region. Malignant transformation is extremely rare and few reports have described its imaging characteristics and prognosis. We present a case showing malignant transformation of EpiC in the left cerebellopontine angle that showed rapid progression 10 years after the initial diagnosis, necessitating craniotomy. Malignant transformation of EpiC was confirmed by pathological examination and whole-body imaging. Based on the present case, we reviewed previously reported cases of malignant transformation of EpiC, identifying 101 cases in the literature. We summarized key aspects such as pathology, extent of resection, postoperative adjuvant therapy, and duration from initial diagnosis to malignant transformation.

## Case report

**Fig. 1:**
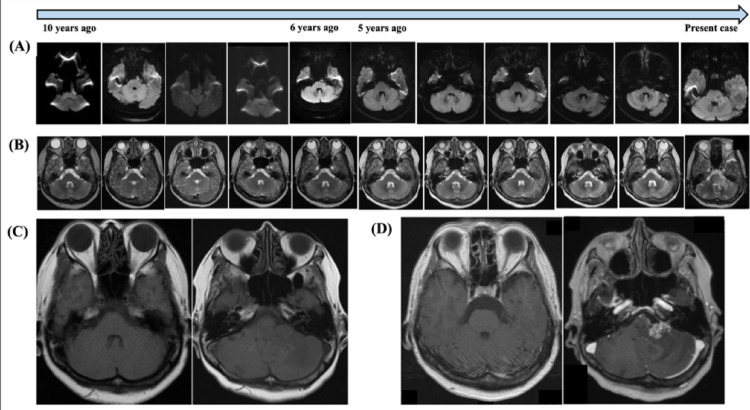
Serial imaging results, **a**,** b)** Serial images from diffusion-weighted imaging (DWI) **(a)** and T2-weighted imaging (T2WI) **(b)** on magnetic resonance imaging (MRI) during 10-year follow-up. Representative slices were selected to best demonstrate the lesion on each imaging study. **c**,** d)** Axial T1-weighted MRI at initial detection **(c)** and just before surgery 10 years later **(d)** (left panels: without Gd enhancement; right panels: with Gd enhancement)

A 69-year-old woman presented with tinnitus that had developed one month earlier. A tumor at the left cerebellopontine angle had been incidentally discovered on magnetic resonance imaging (MRI) a decade earlier, exhibiting high intensity on diffusion-weighted imaging (DWI) and T2-weighted imaging (T2WI) without enhancement on gadolinium-enhanced T1-weighted imaging (T1Gd); these findings were consistent with the typical imaging characteristics of an epidermoid cyst. DWI was performed at the referring hospital with a b-value of 1000 s/mm². Information regarding the MRI scanner manufacturer and magnetic field strength was not available. The case had been followed-up for 10 years with non-contrast MRI during “tumor dormancy status” (Fig. 1a and b). Therefore, we assumed this tumor was benign.

On this presentation, she displayed left facial paralysis and hearing impairment. MRI with gadolinium enhancement was re-investigated, since we suspected that the tumor became malignant progression. Contrast-enhanced lesions appeared within the tumor and left pons, accompanied by edema involving the middle cerebellar peduncle and cerebellar hemisphere (Fig. 1b). In addition, the previously observed hyperintensity on DWI had disappeared (Fig. 1a). There were no tumors in the chest and abdomen CT with serum tumor markers were within normal limits (data not shown).

Due to the rapid symptom progression and the need for pathological confirmation to guide postoperative therapy, surgical resection was performed.

### Intraoperative findings

**Fig. 2:**
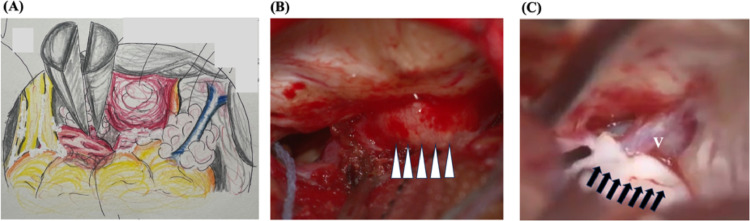
Intraoperative findings for gross appearance of the tumor. Schematic drawing of intraoperative findings of the tumor at the cerebellopontine angle **(a)**. A red component (arrowhead) appears attached to the dura along the petrous bone **(b)** and a pearly tumor (arrow) appears adherent to the petrous vein (V) **(c)**

Craniotomy was performed via a retrosigmoid approach. Intraoperatively, two distinct components were observed at the left cerebellopontine angle (Fig. 2a). The first component was a red-white hemorrhagic tumor wrapped in a membrane adherent to the cerebellum, located near the acoustic meatus and lower cranial nerves (Fig. 2b). The second component was a pearly tumor situated around the petrosal vein, invading the internal acoustic canal and adjacent facial and acoustic nerves (Fig. 2c). The facial and acoustic nerves could not be clearly detached from the tumor, so surgery was ended with partial resection.

## Pathological findings

**Fig. 3:**
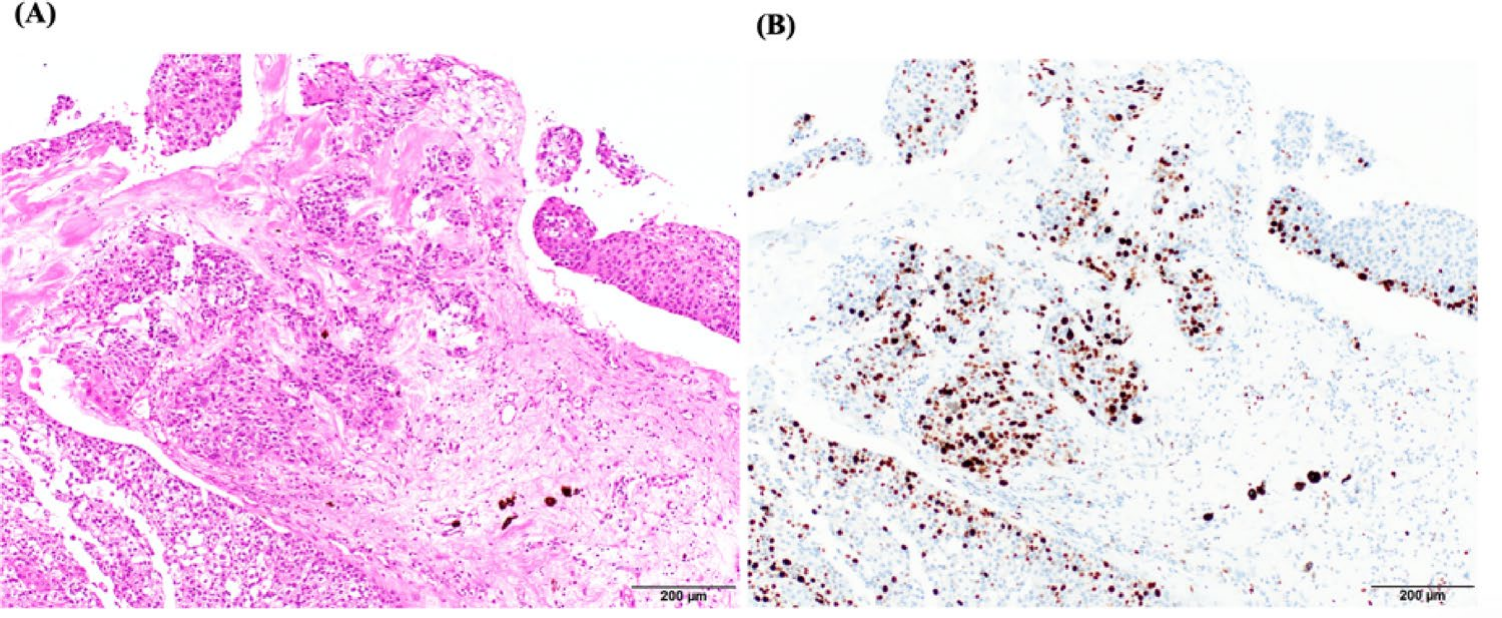
Pathological findings of the tumor at the cerebellopontine angle. **a)** Hematoxylin and eosin staining. Typical squamous cell lineage and keratinization was obsereved. **b)**. Immunohistochemistry for MIB-1. Tumor cells in recurrent tumors were strongly positive for MIB-1 of which labeling index was 50.8%

Atypical epithelial cells with loss of polarity were observed within the membranous structures. In the main tumor mass, nests of squamous cell carcinoma (SCC) cells were found densely proliferating against a background of fibrous stroma, with minimal keratinization but evident necrosis. Typical findings of keratinization for EpiC were not observed (Fig. 3a). The MIB-1 index was 50.8% (Fig. 3b).

## Postoperative clinical course

**Fig. 4:**
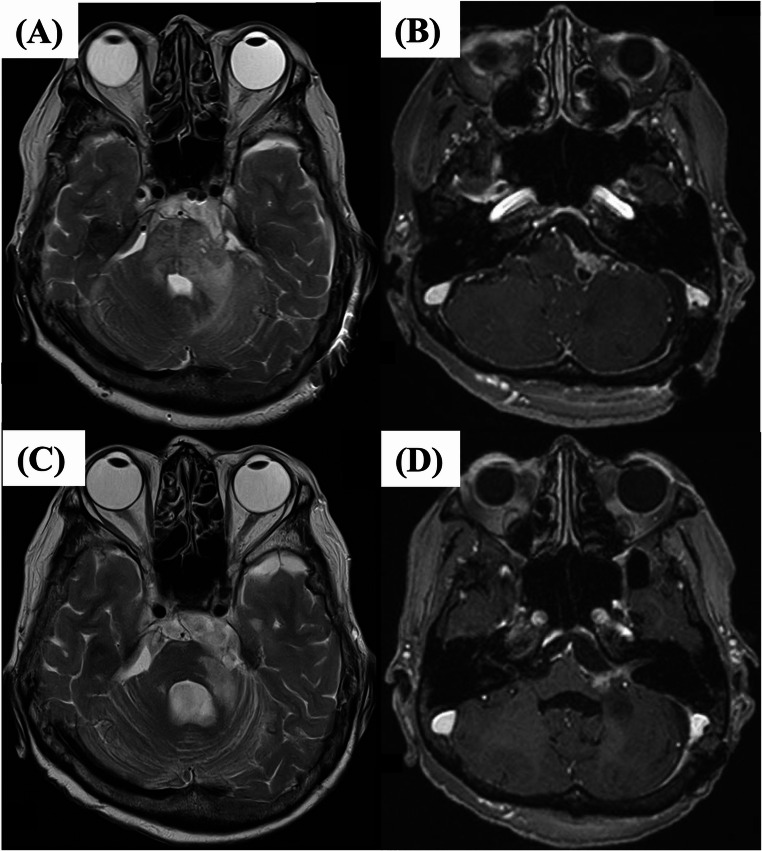
Postoperative magnetic resonance imaging (MRI). T2-weighted **(a)** and T1-weighted imaging with gadolinium enhancement **(b)** showing the residual tumor with high-intensity signal at the middle cerebellar peduncle **(a)** and tumor along the facial nerve **(b)**. MRI after radiotherapy. T2- **(c)** and T1-weighted imaging with gadolinium enhancement **(d)** show regression of the perifocal edema **(c)** and residual tumor **(d)**

Based on the pathological findings as described above, whole-body computed tomography (CT) was performed and tumor marker levels were examined. No primary cancers were identified elsewhere. Given the long-term clinical course detected by radiographic findings along with histological and serological findings, the final diagnosis was SCC representing malignant transformation of EpiC. The patient underwent postoperative local radiotherapy (54 Gy in 27 fractions) to the residual tumor identified on postoperative MRI (Fig. 4a and b). After treatment, the contrast-enhanced lesion and associated edema tended to regress without further progression (Fig. 4c and d). However, 4 months later, the patient gradually developed immobility and was diagnosed with carcinomatous meningitis from lumbar puncture, although no new abnormalities were detected on MRI. The patient was transferred to another facility for palliative care.

## Discussion

We considered two possible mechanisms for the malignant transformation of the EpiC: malignant transformation of residual tumor tissue, or de novo emergence of a malignant tumor. An issue of interest was whether any difference in overall survival exists between cases in which surgery was performed after the development of new symptoms or imaging changes, as in the present case, and cases in which surgery was performed at the time of initial diagnosis. The problem of the present case to diagnose the malignant transformation of EpiC was the lack of initial pathological specimen. We judged by neuroradiological alteration from the non-enhanced tumor to the enhanced tumor with deteriorated clinical symptom.

### Review of EpiC with malignant transformation (Table;1)

**Table 1:** Characteristics of literature included in the review (n=103). In this review, mean age at diagnosis was 54 years, and the most common location was the posterior cranial fossa, including the cerebellopontine angle (76.5%). Mean interval from initial diagnosis to malignant transformation was 105.5 months. Among the 48 reported cases, 38 underwent radiotherapy, 4 underwent chemotherapy alone, and 6 received chemoradiotherapy

Parameter		*n* (%)	Mean
Age, years		101	54
Sex	male	54 (53.5)	
	female	47 (46.5)	
Location	cerebellopontine angle	57 (56.4)	
	other posterior fossa	19 (18.8)	
	supratentorial	23 (22.8)	
Interval between initial & salvage surgery, months		48 (47.5)	106.1
Postoperative adjuvant therapy	radiation	38 (37.6)	
	chemotherapy	4 (4.0)	
	chemoradiation	6 (5.9)	
Pathological samples obtained	initial surgery	94 (93.1)	
	salvage surgery/autopsy	40 * (39.6)	

* Diagnosis determined by CSF cytology (*n*=2)

We reviewed previously reported cases of malignant transformation of EpiC, identifying 103 cases in the literature (Tables 1 and 3). To review previously reported cases of malignant transformation of epidermoid cysts, we investigated a literature search in PubMed using the keywords “epidermoid cyst” and “malignant transformation.” Additional cases changing from EpiC to malignant transformation were identified through manual screening of the reference lists of relevant articles. In this review, mean age at diagnosis was 54 years, and the most common location was the posterior cranial fossa, including the cerebellopontine angle (76.5%). Mean interval from initial diagnosis to malignant transformation was 106.1 months, with the longest reported interval being 480 months [2]. The present case showed a relatively long period of follow-up compared to previous reports. In addition, the true interval in this case may have exceeded 120 months, as the patient was asymptomatic at the time of initial diagnosis.

### Consideration of latent period between initial diagnosis and malignant transformation

**Fig. 5:**
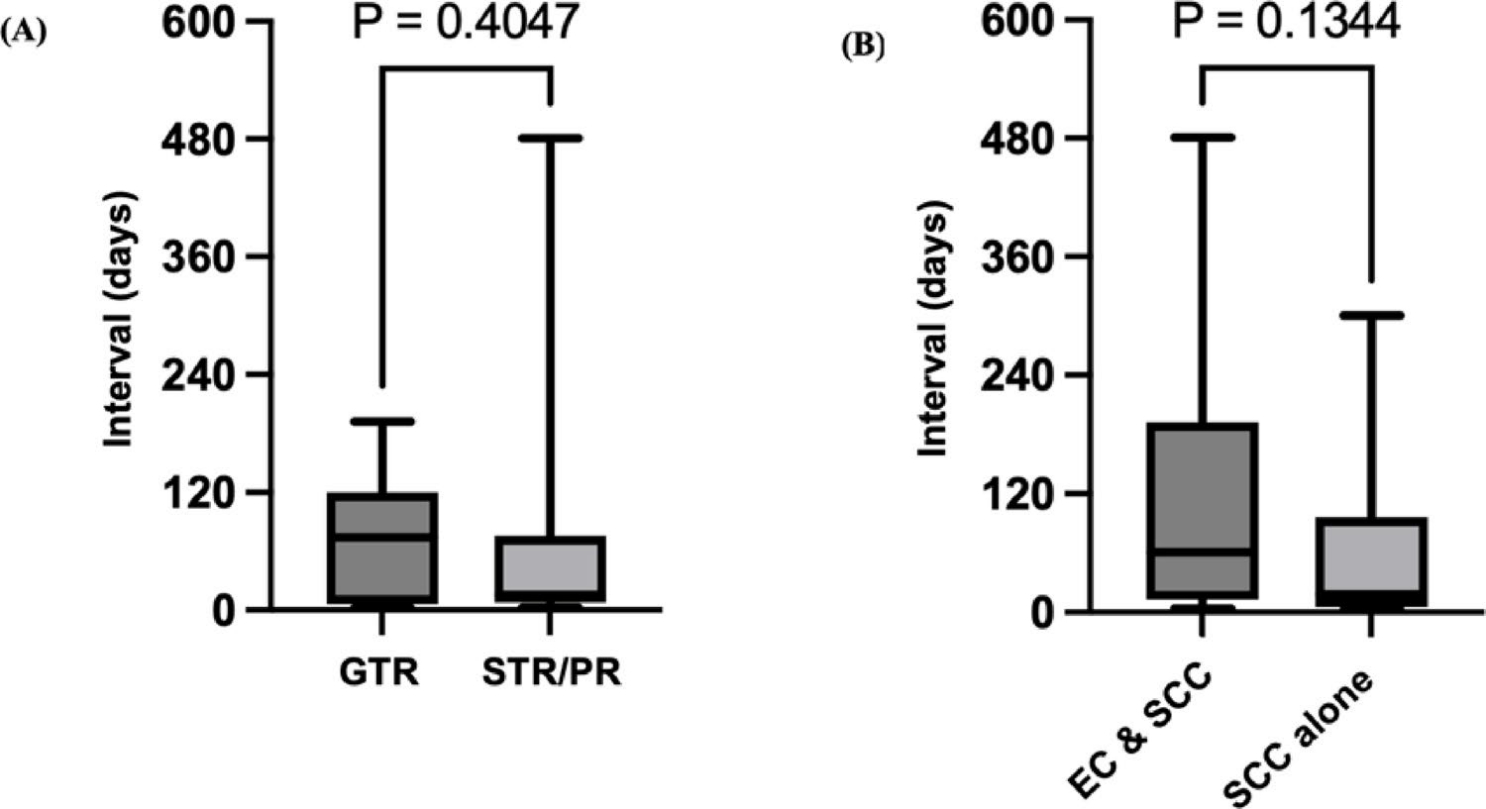
Comparison of interval between initial surgery and salvage surgery for recurrence**. (a)** The interval (in months) tended to be longer for the group with gross total resection (GTR) (*n* = 11) than in the group with subtotal resection (STR) or partial resection (PR) (*n* = 17), although the difference was not significant (*p* = 0.423). **(b)** The interval tended to be longer in the group with concomitant epidermoid cyst and squamous cell carcinoma (EpiC + SCC) (*n* = 19) than in the group with SCC alone (*n* = 14), although the difference was not significant (*p* = 0.120)

Forty-nine cases were included in the comparative analysis of the latent period of malignant transformation, stratified by extent of resection. This included 20 cases classified as gross total resection (GTR) and 29 cases classified as STR or PR. Among these 49 cases, analysis was performed on those cases with a clearly documented interval to recurrence. Mean interval from initial diagnosis to malignant transformation was 79.9 months in the GTR group and 82.1 months in the STR/PR group (GTR, *n* = 11 or STR] or [PR] [*n* = 17]), showing no significant difference (*p* = 0.423; Fig. 5A). Statistical analysis was performed using the Mann–Whitney test. Due to the small number of reported cases and the presence of a few STR/PR cases with exceptionally long intervals, these results should be interpreted with caution [2–4].

### Imaging changes suggestive of malignant transformation

Previous reports have suggested that rapid progression of clinical symptoms, changes in tumor appearance on DWI and T2WI, appearance of contrast-enhanced lesions, and development of peritumoral edema are indicative of malignant transformation [1]. In the present case, a change in DWI findings—specifically, the disappearance of lesion hyperintensity—was observed between 5 and 6 years before surgery, despite the asymptomatic status of the patient (Fig. 1a). No significant changes were observed on T2WI during the same period (Fig. 1b). In the present case, malignant transformation might have occurred during this period, but accurate timing of the malignant transformation was difficult since the patient remained asymptomatic and contrast-enhanced MRI had not been performed during this period. If contrast-enhanced MRI had been conducted, the malignant transformation might have been detected earlier.

### Pathological diagnosis of initial and recurrent tumors (Table 2)

**Table 2:** Pathological diagnoses of initial and recurrent tumors (*n* = 94). Ninety-four cases included information about pathological diagnosis. Among these, 40 cases were diagnosed as EpiC alone, 48 cases as EpiC with SCC (including 10 autopsy cases), and 6 cases as SCC alone. Of these, recurrent tumor samples were available for only 39 cases

Pathological diagnosis	*n*			
	Initial sample		Recurrent sample	
EpiC alone	40		0	
EpiC and SCC	48*		20	
SCC alone	6		20 * *	

Ninety-four cases included information about pathological diagnosis (Table 2). Among these, 40 cases were diagnosed as EpiC alone, 48 cases as EpiC with SCC (including 10 autopsy cases), and 6 cases as SCC alone. Of these, recurrent tumor samples were available for only 40 cases, as the other cases already diagnosed with malignant transformation had proceeded directly to postoperative therapy. Among the recurrent cases, two were diagnosed by lumbar puncture [5, 6], and no initial pathological specimens were available in two other cases; the initial diagnoses in these cases were based on MRI findings, similar to the present case [7, 8]. We analyzed the difference in mean interval from initial diagnosis to second operation between samples of recurrence comprising EpiC with SCC and SCC alone. The mean interval was 121 months in the EpiC with SCC group and 60 months in the SCC alone group (EpiC with SCC [*n* = 19] or SCC alone [*n* = 14]), again showing no significant difference (*p* = 0.120). Statistical analysis was performed using the Mann–Whitney test. This finding may suggest that the ratio of benign to malignant components is associated with the latent period of malignant transformation. Further investigation is required to validate these results (Fig. 5B).

### Significance of concomitant EpiC and SCC (Table 3) [1–102]

To verify malignant transformation of EpiC precisely and rule out the possibility of SCC from occult primary cancers, intraoperative findings might be useful for diagnosing tumors and clarifying whether benign and malignant components co-exist. Only four cases have been reported in which tumors exhibited two distinct components—a red-white hemorrhagic mass and a pearly tumor—similar to the present case. All of these were pathologically diagnosed as EpiC with SCC. Although the coexistence of two components does not establish a direct causal relationship, these findings suggest the possibility of malignant transformation of EpiC. Pathological diagnosis is essential for understanding the biological characteristics of a tumor and evaluating the growth potential, both of which are critical for predicting overall survival and making decisions regarding postoperative adjuvant therapy.

**Table 3:** Lists of patients with epidermoid cyst with malignant transformation in the literature review (*n* = 103). CPA, cerebellopontine angle; EpiC, epidermoid cyst; F, female; GTR, gross total resection; M, male, n.d; not described; RT, radiotherapy; SCC, squamous cell carcinoma; STR, subtotal resection

Reference	Sex	Age (Years)	Histopathology at initial surgery	Interval from initial surgery to recurrence (months)	Histopathology at surgery for recurrence	Extent of resection	Location	Adjuvant therapy	Follow-up	Immunohistochemistry, MIB-1 index
Narasimhaiah D et al. 2023 [11]	M	39	EpiC	5	SCC	GTR	CPA	n.d.	n.d.	p63(+), MIB-1 40%
Gabay S et al. 2022 [37]	M	61	EpiC+SCC	n.d.	n.d.	STR	CPA	RT, remission at 2 months	13 months	n.d.
Mliyh L et al. 2023 [70]	F	80	EpiC+SCC	n.d.	n.d.	STR	CPA	n.d.	n.d.	p16(-)
Zhang X et al. 2023 [102]	M	58	EpiC+SCC	n.d.	n.d.	GTR	supratentorial	RT, no recurrence at 11 months	n.d.	n.d.
Yang T et al. 2024 [14]	M	61	EpiC+SCC	n.d.	−	GTR	CPA	chemoradiation	11 months	CK (AE1/AE3)(+),
										GATA3(+), vimentin(+),
										MIB-1 20%
Dey S et al. 2024 [28]	F	52	SCC	n.d.	−	STR	dorsal midbrain	RT	n.d.	n.d.
Fox H et al. 1965 [35]	M	50	EpiC	84	EpiC+SCC	STR	temporal lobe	n.d.	n.d.	n.d.
Toglia JU et al. 1965 [98]	F	53	EpiC	12	EpiC+SCC	STR	skull base	n.d.	died at 1 day	n.d.
Goldman SA et al. 1987 [3]	F	59	EpiC	396	EpiC+SCC	STR	lateral ventricle	RT	36 months	n.d.
Salazar et al. 1987 [87]	M	49	EpiC	3	EpiC+SCC	GTR or STR	CPA	n.d.	none	n.d.
Abramson RC et al. 1989 [15]	M	37	EpiC	60	SCC	GTR	CPA	n.d.	9 months	n.d.
Nishiura I et al. 1989 [78]	M	38	EpiC	6	EpiC+SCC	STR	CPA	chemotherapy	stable at 24 months	n.d.
Knorr JR et al. 1991 [55]	M	74	EpiC	13	EpiC+SCC	GTR or STR	CPA	n.d.	n.d.	n.d.
Tognetti F et al. 1991 [97]	F	67	EpiC	372	−	N	frontotemporal	n.d.	n.d.	n.d.
Bayindir C et al. 1996 [20]	F	67	EpiC	10	SCC	STR	lateral ventricle	none	36 months	n.d.
Murase S et al. 1999 [73]	F	40	EpiC	120	SCC	GTR	CPA	chemotherapy	stable at 60 months	n.d.
Asahi T et al. 2001 [9]	F	55	EpiC	156	EpiC+SCC	GTR	CPA	n.d.	died at 3 months	EMA(+), MIB-1 30%
Link M et al. 2002 [64]	F	57	EpiC	12	EpiC+SCC	STR	CPA	RT	died immediately	n.d.
Akar Z et al. 2003 [18]	F		EpiC	18	SCC	STR	CPA	n.d.	died at 5 months	n.d.
Hamlat A et al. 2005 [44]	F	62	EpiC+SCC	10	−	N	temporal lobe	chemotherapy	died at 7 months	n.d.
Tamura K et al. 2006 [96]	F	56	EpiC	96	SCC	GTR	CPA	RT	18 months	n.d.
Ge P et al. 2009 [39]	M	50	EpiC	74	EpiC+SCC	GTR	temporal lobe	n.d.	N	n.d.
Kano T et al. 2010 [4]	F	64	EpiC	192	SCC	STR	CPA	RT	died at 36 months	n.d.
Nakao Y et al. 2010 [75]	F	74	EpiC	240	EpiC+SCC	GTR or STR	CPA	RT	17 months	n.d.
Hao S et al. 2010 [7]	F	61	EpiC	72	EpiC+SCC	STR	CPA	n.d.	died at 1.5 months	n.d.
Lakhdar F et al. 2011 [61]	M	52	EpiC	6	EpiC+SCC	GTR	CPA	RT	n.d.	n.d.
Chon K et al. 2012 [24]	M	43	EpiC	5	SCC	STR	CPA	RT	none	n.d.
Vellutini E et al. 2014 [100]	F	42	EpiC	24	EpiC+SCC	STR	CPA	n.d.	n.d.	n.d.
Pikis and Margolon 2016 [83]	M	77	EpiC	9	EpiC+SCC	STR	CPA	chemoradiation	died at 6 months	n.d.
Ding S et al. 2016 [29]	F	55	EpiC	7	SCC	STR	temporal lobe	n.d.	none	n.d.
Solanki S et al. 2017 [92]	F	47	n.d.	36	−	N	CPA	n.d.	n.d.	n.d.
Sakamoto H et al. 2021 [1]	F	59	EpiC	108	EpiC+SCC	GTR	CPA	RT	7 months	p53(+), p16(+)
Ernst P et al. 1912 [32]	M	52	EpiC+SCC	n.d.	−	autopsy	CPA	n.d.	n.d.	n.d.
Hug O et al. 1942 [48]	M	42	EpiC+SCC	n.d.	−	autopsy	parapontine	n.d.	n.d.	n.d.
Henkel H et al. 1951 [46]	M	49	EpiC+SCC	n.d.	−	autopsy	parapontine	n.d.	n.d.	n.d.
Yamanaka A et al. 1955 [101]	M	57	EpiC+SCC	n.d.	−	autopsy	skull base	n.d.	n.d.	n.d.
Landers J et al. 1960 [62]	F	73	EpiC+SCC	n.d.	−	autopsy	cerebellum	n.d.	n.d.	n.d.
Kömpf D et al. 1977 [58]	F	57	EpiC+SCC	n.d.	−	autopsy	parapontine	n.d.	n.d.	n.d.
Scully R et al. 1977 [89]	M	59	EpiC+SCC	n.d.	−	GTR or STR	CPA	RT	n.d.	n.d.
Dubois P et al. 1981 [30]	M	53	EpiC+SCC	n.d.	−	STR	CPA	RT	died at 2 months	n.d.
Takado M et al. 1982 [95]	F	54	EpiC+SCC	n.d.	−	biopsy	parapontine	n.d.	n.d.	n.d.
Lewis A et al. 1983 [63]	F	53	EpiC+SCC	n.d.	−	STR	parasellar	n.d.	died at 1.2 months	n.d.
Bondeson L et al. 1984 [21]	F	56	EpiC+SCC	n.d.	−	biopsy	CPA	n.d.	n.d.	n.d.
Giangaspero F et al. 1984 [42]	M	45	SCC	n.d.	−	STR	parietooccipital lobe	RT	n.d.	n.d.
Gi H et al. 1990 [41]	M	39	EpiC+SCC	n.d.	−	GTR	CPA	RT	n.d.	n.d.
Mori Y et al. 1995 [72]	M	41	EpiC+SCC	n.d.	−	STR	CPA	RT	11 months	n.d.
Nishio S et al. 1995 [77]	M	57	EpiC+SCC	n.d.	−	STR	CPA	RT	30 months	n.d.
Kahn E et al. 1955 [52]	F	37	EpiC+SCC	n.d.	n.d.	n.d.	frontal lobe	n.d.	n.d.	n.d.
Davidson S et al. 1960 [26]	M	46	EpiC+SCC	n.d.	n.d.	GTR	frontal lobe	RT	none	n.d.
Komjatszegi S et al. 1964 [57]	F	45	EpiC+SCC	n.d.	n.d.	autopsy	basal	n.d.	n.d.	n.d.
Nosaka Y et al. 1979 [79]	M	46	EpiC+SCC	n.d.	n.d.	STR	CPA	n.d.	died at 7 months	n.d.
Maffazoni D et al. 1986 [65]	M	45	EpiC+SCC	n.d.	−	none	basal	n.d.	n.d.	n.d.
Ishimatsu T et al. 1988 [50]	M	40	EpiC+SCC	n.d.	−	none	CPA	n.d.	n.d.	n.d.
Mohanty V et al. 1996 [71]	M	20	EpiC+SCC	n.d.	−	none	posterior fossa	n.d.	n.d.	n.d.
Ishikawa S et al. 2000 [49]	M	65	EpiC+SCC	n.d.	−	autopsy	CPA	n.d.	n.d.	n.d.
Khan R et al. 2001 [53]	M	53	EpiC+SCC	n.d.	−	autopsy	prepontine	n.d.	n.d.	n.d.
Shirabe T et al. 2003 [91]	M	49	EpiC	n.d.	SCC	biopsy	brainstem	n.d.	died at 24 months	n.d.
Kodama H et al. 2007 [56]	M	67	EpiC	2	SCC	STR	CPA	RT	died at 13 months	n.d.
Pagni F et al. 2007 [81]	F	65	EpiC+SCC	n.d.	SCC (CSF)	biopsy	pineal	n.d.	none	n.d.
Ozuneiz C et al. 2017 [8]	M	64	EpiC(neuroimage)	276	SCC	N	lateral ventricle	n.d.	3 months	n.d.
Michael L et al. 2005 [68]	M	45	EpiC+SCC	n.d.	−	STR	posterior fossa	RT	died immediately	n.d.
Nagata K et al. 2019 [74]	F	77	SCC +(EC? )	n.d.	−	STR	CPA	RT	n.d.	n.d.
Feng R et al. 2014 [33]	M	42	EpiC+SCC	n.d.	−	GTR	CPA	RT	6 months	n.d.
Garcia C et al. 1981 [38]	M	61	SCC	n.d.	EpiC+SCC (autopsy)	biopsy	CPA	RT	died at 6 months	n.d.
Fuse T et al. 1995 [36]	F	74	n.d.	n.d.	−	GTR or STR	CPA	RT	n.d.	n.d.
Uchino A et al. 1995 [99]	M	57	EpiC+SCC	n.d.	−	STR	CPA	RT	4 months	n.d.
Park and Park et al. 2003 [82]	M	65	EpiC+SCC	n.d.	−	STR	CPA	RT	none	n.d.
Guan L et al. 2004 [43]	F	42	EpiC	204	−	GTR or STR	temporal lobe	RT	n.d.	n.d.
Kim and Kim et al. 2008 [54]	F	72	EpiC+SCC	n.d.	−	STR	CPA	RT	12 months	n.d.
Roh T et al. 2017 [86]	F	53	EpiC+SCC	n.d.	−	GTR	CPA	RT	n.d.	n.d.
Chen Z et al. 2019 [23]	M	43	EpiC+SCC	n.d.	−	STR	CPA	RT	24 months	AE1/3(+)
Fereydonyan N et al. 2019 [34]		30	EpiC	60	EpiC+SCC	GTR	CPA	RT	24 months	n.d.
Gerges M et al. 2019 [40]	F	65	EpiC+SCC	n.d.	−	GTR	pineal	RT	6 months	n.d.
Badat N et al. 2018 [19]		70	n.d.	n.d.	−	N	CPA	n.d.	n.d.	n.d.
Kadashev B et al. 2003 [51]	F	47	EpiC+SCC	n.d.	−	GTR or STR	temporal lobe	n.d.	n.d.	n.d.
Sawan B et al. 2000 [88]	M	66	EpiC+SCC	n.d.	−	autopsy	prepontine	n.d.	n.d.	n.d.
Chourmouzi D et al. 2015 [25]	F	39	EpiC+SCC	n.d.	−	GTR	CPA	n.d.	none	n.d.
Seif B et al. 2017 [90]	M	83	EpiC	2	SCC	GTR	CPA	n.d.	none	n.d.
Alsadi H et al. 2025 [5]	M	59	EpiC	168	SCC (CSF)	n.d.	CPA	chemotherapy	died at 2 months	n.d.
Agarwal S et al. 2007 [17]	M	45	EpiC+SCC	n.d.	−	N	posterior fossa	RT	lost	n.d.
Elsarraj H et al. 2025 [6]	M	61	EpiC	156	SCC (CSF)	STR	CPA	chemotherapy	n.d.	n.d.
Michenet P et al. 1989 [69]	M	56	EpiC	192	EpiC+SCC	GTR	n.d.	n.d.	n.d.	n.d.
Hoeffel C et al. 1997 [47]	M	43	EpiC+SCC	n.d.	−	N	parietal and occipital bone	chemoradiation	n.d.	n.d.
Raheja A et al. 2016 [85]	F	54	EpiC+SCC	n.d.	−	STR	prepontine	n.d.	n.d.	n.d.
Raheja A et al. 2016 [85]	F	37	EpiC+SCC	n.d.	−	biopsy	prepontine	n.d.	n.d.	n.d.
Mascarenhas A et al. 2017 [66]	F	35	EpiC	60	EpiC+SCC	STR	CPA	n.d.	n.d.	n.d.
Cuoco J et al. 2019 [2]	M	71	EpiC	480	EpiC+SCC	STR	CPA	RT	none	CK5/6(+)
Radhakrishnan V et al. 1994 [84]	M	53	EpiC	372	EpiC+SCC	GTR or STR	frontal	n.d.	n.d.	n.d.
Song Z et al. 2024 [12]	F	47	EpiC+SCC	n.d.	−	GTR	basal ganglia and thalamus	chemoradiation	n.d.	CK5/6(+), P63(+),
										β-catenin(+), MIB-1 40%
Acciari N et al. 1993 [16]			SCC+(EC? )	n.d.	−	N	chiasmatic-parasellar	n.d.	n.d.	n.d.
Bretschneider T et al. 1999 [22]	F	71	EpiC+SCC	n.d.	−	STR	occipital bone	RT	died at 13 months	n.d.
Eatz T et al. 2023 [31]	F	43	n.d.	n.d.	n.d.	n.d.	CPA	n.d.	n.d.	n.d.
Kubokura T et al. 1986 [59]	F	60	EpiC+SCC	n.d.	n.d.	STR	suprasellar and temporal	n.d.	died at 6 days	n.d.
Matsuno A et al. 1987 [67]	M	43	EpiC	7	n.d.	STR	CPA	chemoradiation	n.d.	n.d.
Delangre T et al. 1992 [27]	F	72	EpiC+SCC	n.d.	n.d.	n.d.	CPA	n.d.	n.d.	n.d.
Nawashiro H et al. 2001 [76]	M	46	n.d.	n.d.	n.d.	n.d.	temporal lobe	n.d.	n.d.	n.d.
Hatem O et al. 2002 [45]	M	40	n.d.	n.d.	n.d.	n.d.	frontotemporal prepontine	RT	n.d.	n.d.
Kwon S et al. 2019 [60]	M	71	n.d.	n.d.	n.d.	STR	n.d.	n.d.	n.d.	n.d.
Ou A et al. 2018 [80]	F	71	EpiC	300	SCC	GTR or STR	CPA	RT	n.d.	n.d.
Suematsu Y et al. 2018 [94]	M	54	EpiC	24	SCC	GTR or STR	CPA	n.d.	n.d.	n.d.
Monaco R et al. 2003 [10]	M	36	EpiC+SCC	n.d.	−	GTR	posterior fossa	n.d.	24 months	AE1/3(+), MIB-1 80%
Sun T et al. 2016 [13]	F	22	EpiC+SCC	n.d.	SCC	STR	CPA	RT	reoperation at 19 months,	CK5/6(+), MIB-1 60%
									died at 41 months	
Sterner R et al. 2024 [93]	F	63	EpiC	6	SCC	GTR or STR	orbit	n.d.	n.d.	n.d.

CPA, cerebellopontine angle; EpiC, epidermoid cyst; F, female; GTR, gross total resection; M, male

n.d., not described; RT, radiotherapy; SCC, squamous cell carcinoma; STR, subtotal resection

In six cases, the MIB-1 labeling index was found to be high (range, 20–80%) [13, 67, 72, 90, 93, 101]. In the present case, consistent with previous reports, the MIB-1 index was 50.8%, suggesting a high proliferative potential of the tumor cells. Despite a lack of histological confirmation of EpiC during the clinical course of the present case, we assumed the tumor represented a malignant transformation from EpiC rather than SCC metastasis from an unknown primary cancer, as evidenced by serial MRI during long-term follow-up, especially the first 5 years of dormancy as shown in T2WI and DWI (Fig. 1a and b). Even during the most recent five years when radiological findings from T2WI and DWI changed, growth potential was not rapid, representing an obvious difference from the typical behavior of SCC metastasis.

### Role of postoperative adjuvant therapy for SCC at recurrence (Table 3)

In cases of malignant transformation, the issue of whether to perform radiotherapy or chemotherapy in addition to surgery is important. However, no standard treatment strategy has been established. Among the 48 reported cases, 38 underwent radiotherapy, 4 underwent chemotherapy alone, and 6 received chemoradiotherapy. In addition, follow-up periods remain short and treatment regimens have not been standardized or clearly described in detail. In the present case, the contrast-enhanced lesion disappeared following local radiotherapy, suggesting that radiotherapy may be effective. Malignant transformation of EpiC is extremely rare, making a definitive algorithm for postoperative therapy difficult to establish. EpiC is recognized as a benign tumor and malignant transformation is rare, so long-term follow-up is essential for identifying malignant transformation through imaging and pathological evaluation.

### Limitations

In the present case, whether the tumor consisted solely of EpiC in the initial stage was unclear, as no initial surgery was performed. In addition, pathological examination revealed only SCC, despite the intraoperative observation of a pearly tumor. Ultimately, malignant transformation was diagnosed based on the initial imaging and intraoperative findings. If pathological changes had been captured through multiple surgeries performed at the time of initial diagnosis and during malignant transformation, or if surgery had been performed immediately after malignant transformation was suspected and features of the transition from EpiC to SCC had been identified, the exact timing of malignant transformation might have been able to be determined. In addition, in the present case, only partial resection was performed, which may have contributed to the inability to detect EpiC components in the submitted specimens. Although no disease progression was observed for 4 months after radiotherapy, the period of follow-up for postoperative therapy remains short, similar to previous reports. Further accumulation of postoperative therapy cases and long-term follow-up are required to validate these observations.

## Conclusions

We experienced malignant transformation of EpiC during the clinical course. Long-term follow-up is essential for identifying such changes, as malignant transformation typically requires an extended period. In particular, if high intensity on DWI disappears or a contrast-enhanced lesion appears, malignant transformation of EpiC should be considered. Postoperative radiotherapy may be effective for controlling residual tumor, but clear evidence remains lacking.

## Abbreviations

DWI: Diffusion-Weighted Imaging
EpiC: Epidermoid Cyst
GTR: Gross Total Resection
MRI: Magnetic Resonance Imaging
PR: Partial Resection
SCC: Squamous Cell Carcinoma
STR: Subtotal Total Resection
T1Gd: Gadolinium-Enhanced T1-Weighted Imaging
T1WI: T1-Weighted Imaging
T2WI: T2-Weighted Imaging
